# An Improved Biomimetic Beaver Behavior Optimizer for Inverse Kinematics of Rehabilitation Robotic Arms

**DOI:** 10.3390/biomimetics11040259

**Published:** 2026-04-08

**Authors:** Shuxin Fan, Yonghong Deng, Zhibin Li

**Affiliations:** 1 Department of Mechanical and Electrical Engineering, Liaocheng University Dongchang College, Liaocheng 252000, China; 2 Key Laboratory of High Performance Scientific Computation, School of Science, Xihua University, Chengdu 610039, China; 3 School of Software Engineering, Chengdu University of Information Technology, Chengdu 610225, China; lizhibin@cuit.edu.cn; 4 Dazhou Key Laboratory of Government Data Security, Sichuan University of Arts and Science, Dazhou 635000, China

**Keywords:** biomimetic optimization, beaver-inspired computation, inverse kinematics, rehabilitation robotics, joint-constrained optimization

## Abstract

Accurate inverse kinematics for rehabilitation robotic arms remains challenging because of strong nonlinearity, multiple feasible joint configurations, and strict joint-limit constraints. Inspired by the cooperative construction, adaptive exploration, and collective information-sharing behaviors of beavers, this study develops an improved biomimetic beaver behavior optimizer (IBBO) for optimization-based inverse kinematics solving. In the proposed framework, biologically inspired cooperative search is translated into an engineering-oriented numerical strategy through four complementary mechanisms: a strict elitist replacement with rollback to preserve population fitness consistency, a momentum-inspired information transfer scheme to accumulate effective search directions, a lightweight memetic coordinate-wise local search to strengthen late-stage exploitation, and an adaptive builder–disturbance schedule to progressively shift the search from exploration to refinement. The optimization capability of IBBO is first evaluated on the CEC2017 benchmark suite, where it demonstrates competitive accuracy and robustness. It is then applied to inverse kinematics solving for representative rehabilitation robotic arms by minimizing pose errors under joint constraints. The experimental results show that IBBO can consistently generate feasible joint solutions with improved terminal pose accuracy and stable convergence compared with baseline metaheuristics. Beyond numerical improvement, this study provides a biomimetic optimization framework that transfers beaver-inspired cooperative behaviors into rehabilitation robotics, offering an effective computational approach for constrained inverse kinematics problems.

## 1. Introduction

Stroke is widely recognized as a leading cause of long-term disability worldwide. A large proportion of stroke survivors experience early upper-limb motor impairment, which can substantially reduce independence in activities of daily living and negatively affect quality of life, while also imposing considerable caregiving demands and socioeconomic burden [1,2,3]. Clinical and experimental evidence suggests that intensive, repetitive, and task-oriented rehabilitation training can promote neuroplasticity and facilitate the recovery of voluntary muscle control, thereby improving upper-limb function [4]. However, conventional therapist-led rehabilitation is often limited by workforce capacity and treatment variability, and it can be difficult to deliver highly consistent, high-dose training over sustained periods [5]. With advances in medical robotics and human–robot interaction, upper-limb rehabilitation robots offer a promising approach to provide high-intensity, repeatable, and precisely controlled training, enabling standardized therapy delivery and supporting functional recovery through structured, patient-specific exercise programs [6].

In rehabilitation robotics, inverse kinematics is frequently required for real-time motion generation, assist-as-needed control, and trajectory tracking of upper-limb robotic arms or exoskeleton devices. Unlike many conventional industrial manipulators that are designed around kinematic forms amenable to analytical inverse kinematics, rehabilitation platforms often adopt patient-centric and ergonomics-driven architectures, including redundant degrees of freedom, nonstandard joint layouts, and adjustable link parameters to accommodate inter-subject variability [7]. These design characteristics, together with manufacturing tolerances and assembly-induced parameter deviations, can make closed-form inverse kinematics either unavailable or overly sensitive to model uncertainties. Consequently, numerical inverse kinematics remains the predominant approach, yet classical Jacobian-based iterative schemes may suffer from poor conditioning in the vicinity of singular or near-singular configurations, which can degrade convergence reliability and induce large, undesirable joint excursions [8,9]. To enhance robustness under joint limits and human-centered constraints, an increasingly common strategy is to formulate inverse kinematics as a constrained optimization problem that directly minimizes end-effector pose discrepancy while incorporating feasibility requirements such as range-of-motion bounds, comfort-related penalties, and smoothness regularization. This optimization perspective provides a flexible computational basis for integrating intelligent or metaheuristic solvers, which can improve global search capability and reduce sensitivity to initial guesses when addressing nonlinear, multimodal inverse kinematics in rehabilitation robotic applications [10].

To enable efficient inverse kinematics computation for general purpose robots across arbitrary target poses, recent studies increasingly adopt intelligent optimization methods. The core approach is to reformulate the robot’s kinematic constraints as an optimization-based control problem, and then obtain feasible joint configurations by solving this problem numerically. Many researchers have extensively studied this topic. For example, Khoramshahi et al. [11] proposed a task-informed framework based on a kinematic model that leverages the weighted Jacobian pseudo-inverse and its null space to quantify coordination and estimate inverse kinematics weights directly from observed kinematic data. Ning et al. [12] addressed the inverse kinematics of redundant upper-limb exoskeleton rehabilitation robots by formulating a multi-objective optimization model that integrates end-effector pose tracking, joint-motion comfort, energy consumption, motion safety, and human-like constraints, and demonstrated that an improved equilibrium optimization solver can achieve fast, accurate, and robust inverse kinematics solutions while significantly improving the human–robot motion–shape compatibility through both discrete and continuous trajectory experiments. Zhao et al. [13] addressed robot inverse kinematics by proposing an improved PSO with adaptive inertia weight adjustment and joint limit-based constraints, and solved the inverse kinematics. Slim et al. [14] proposed an intrinsically modified bat algorithm to solve robotic-arm inverse kinematics by embedding a joint-elongation minimization mechanism into the bat update rules, thereby obtaining inverse kinematics solutions with minimal joint variation from the initial configuration and demonstrating effectiveness on both benchmark comparisons and a real spherical-wrist manipulator. Chagas et al. [15] developed an optimization algorithm for humanoid robot walking based on an inverse kinematics model integrated with a genetic algorithm, aiming to enhance sagittal displacement and minimize lateral deviation during locomotion, and validated its effectiveness through both a virtual simulator and a humanoid robotic platform. Liu et al. [16] proposed an improved particle swarm optimization algorithm for solving the inverse kinematics of general robots that do not satisfy the Pieper criterion, introducing a nonlinear dynamic inertia weight adjustment strategy based on similarity to enhance robustness, and a multi-population strategy with an immigration operator to increase diversity, which demonstrated superior stability, accuracy, and convergence speed compared to standard PSO and multi-subswarm methods. Duymazlar et al. [17] proposed a swarm intelligence-based boomerang algorithm with a recursive structure for solving the inverse kinematics of robot arms, aiming to reduce computation time without sacrificing accuracy, and demonstrated its superiority over PSO variants through simulations and experiments. Although various studies have achieved progress in solving robot inverse kinematics, the motion model of a robot is inherently a high-dimensional, nonlinear, and multi-modal problem. As the degrees of freedom increase, traditional algorithms often face difficulties such as premature convergence, local optima entrapment, and reduced solution stability. These challenges highlight the need for more robust and adaptive optimization methods.

From the perspective of classical inverse kinematics theory, non-uniqueness in redundant manipulators is commonly addressed by Jacobian pseudo-inverse, damped least-squares, or null-space projection methods, where secondary objectives such as joint-limit avoidance, posture regularity, or comfort can be embedded in the null space. However, the present study focuses on the optimization-based inverse kinematics of the specific rehabilitation mechanism considered here, in which the active joint variables are solved under strict joint bounds and the passive DOFs are not treated as optimization variables. Therefore, the main emphasis of this work is on constrained multi-solution inverse kinematics solving rather than explicit null-space redundancy resolution.

To address the challenges posed by high-dimensional, nonlinear, and multi-solution characteristics in robotic inverse kinematics, this paper proposes an enhanced beaver behavior optimizer (IBBO) that integrates four complementary strategies: a strict elitist replacement with rollback to maintain population consistency; a momentum-driven information transfer mechanism to accelerate convergence; a lightweight memetic coordinate-wise search with adaptive step control for refined exploitation; and an adaptive disturbance scheduling scheme to balance exploration and exploitation throughout the optimization process. The main innovations of this work are twofold:

(a) A biomimetic optimization framework for rehabilitation robotic-arm inverse kinematics is established by abstracting cooperative and adaptive beaver behaviors into an engineering-oriented numerical solver.
(b) An improved beaver behavior optimizer is developed by integrating strict elitist replacement with rollback, momentum-inspired information transfer, a lightweight memetic local search, and an adaptive builder–disturbance schedule to strengthen convergence reliability and terminal accuracy.
(c) The proposed framework is validated on both CEC2017 benchmark functions and representative rehabilitation robotic-arm inverse kinematics tasks, demonstrating improved solution accuracy, robustness, and practical feasibility under joint constraints.

The main structure of this paper is organized as follows: Section 2 introduces the inverse kinematics model of the rehabilitation robotic arm. Section 3 presents the proposed enhanced beaver behavior optimizer for optimization-based inverse kinematics. Section 4 provides experimental validation and results analysis, and Section 5 concludes with the conclusions and future work.

## 2. Inverse Kinematics Model of the Rehabilitation Robot

**Figure 1:**
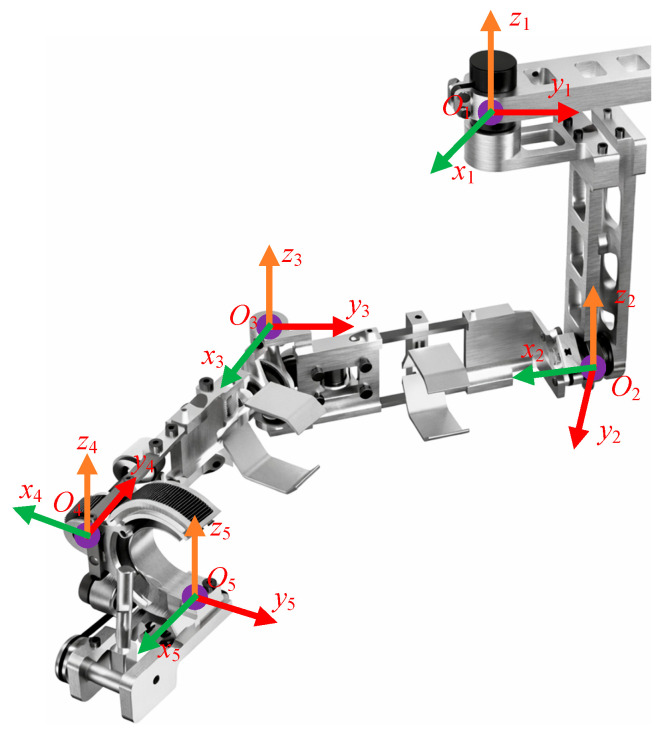
Structure of the rehabilitation robot and its coordinate system.

As shown in Figure 1, the upper-limb rehabilitation exoskeleton has a three-dimensional configuration with five active degrees of freedom (DOFs) and two passive DOFs. The five active DOFs correspond to the joint motions illustrated in Figure 1 and provide the primary posture and displacement control required for rehabilitation training. In the present inverse kinematics formulation, only these five active joints are treated as decision variables, whereas the two passive DOFs mainly serve as compliance-enhancing mechanical accommodations and are not explicitly optimized. Both the upper-arm and forearm modules provide an adjustable travel of approximately 0.050 m to accommodate users with different body sizes. At the elbow, passive compliance is realized through a bevel-gear pair. Specifically, the large bevel gear is connected to a short shaft that drives the main forearm assembly to follow forearm rotation, thereby enabling compliant motion. The small bevel gear is mounted on a bushing around the elbow shaft and does not rotate coaxially with it; however, it remains continuously engaged with the large gear, which improves transmission stability and connection reliability. In practical use, maintaining real-time alignment between the instantaneous rotation center of the shoulder mechanism and the anatomical shoulder joint center is challenging. Such misalignment may lead to human–robot kinematic incompatibility, discomfort, and potential safety risks. To mitigate this issue, a shoulder slider mechanism is incorporated to finely adjust the position of the instantaneous shoulder axis. Moreover, the two passive DOFs further enhance the motion compliance of the exoskeleton, thereby improving training adaptability and increasing the comfort of the affected limb.

After establishing the D-H coordinate model of the upper-limb rehabilitation robot, the obtained D-H parameters are used to derive the homogeneous pose transformation matrices between adjacent links. By sequentially multiplying these matrices along the kinematic chain, the pose of the end-effector can be expressed. To facilitate the definition of link coordinate frames, the robot model is appropriately simplified by equivalently consolidating the two shoulder-joint degrees of freedom at point *O*_1_. The resulting modified D-H coordinate system is illustrated in Figure 1.

The corresponding D-H parameters are characterized by four quantities: the link length *a_i_*, link twist *α_i_*, link offset *d_i_*, and joint angle *θ_i_*. The resulting D-H parameter set for the upper-limb rehabilitation robot is summarized in Table 1.

According to the Chinese national standard GB 10000–88 (Anthropometric data of Chinese adults), the typical forearm length of adults falls within 185–268 mm, while the upper-arm length is generally in the range of 252–349 mm. Considering that the proposed upper-limb rehabilitation robot provides an adjustable travel of ±50 mm for both the forearm and upper-arm modules, the link dimensions were selected to cover the target population while retaining a sufficient adjustment margin. Specifically, the forearm-related link length *d*_4_ was set to 235 mm, the upper-arm-related link length *a*_2_ was set to 310 mm, and the link length *a*_4_ was chosen as 100 mm.

In the D-H formulation, a sequence of coordinate frames {*i*} is assigned along the kinematic chain, where the *Z_i_* axis is aligned with the axis of joint iii. For two consecutive frames {*i*−1} and {*i*}, the link length *a_i_*_−1_ is defined as the distance from the *Z_i_*_−1_ axis to the *Z_i_* axis measured along the *X_i_*_−1_ direction, and the link twist *α_i_*_−1_ is the angle that rotates *Z_i_*_−1_ into *Z_i_* about *X_i_*_−1_. The link offset *d_i_* denotes the signed displacement along *Z_i_*_−1_ from the origin of frame {*i*−1} to the point where the common normal intersects the joint axis, while the joint variable *θ_i_* represents the rotation from *X_i_*_−1_ to *X_i_* about *Z_i_*_−1_. With these definitions, the homogeneous transformation between adjacent frames can be constructed, and the end-effector pose is obtained by successive multiplication of the transformations.

Although swarm and evolutionary optimizers have been widely employed to solve inverse kinematics in a derivative-free manner, the inverse kinematics of rehabilitation robotic arms remain a high-dimensional and strongly nonlinear problem with multiple feasible joint configurations and strict joint-limit constraints. As the degrees of freedom increase and the feasible region becomes narrow, metaheuristic solvers frequently encounter stagnation or premature convergence, which degrades terminal pose accuracy and makes convergence behavior difficult to guarantee. To alleviate these issues, this paper develops an IBBO for optimization-based inverse kinematics, where four complementary mechanisms are integrated to enhance solution precision and robustness, including elitist replacement with rollback, momentum-guided information transfer, a lightweight coordinate-wise memetic local search, and an adaptive disturbance scheduling strategy.

Based on the definitions above, the homogeneous transformation from frame {*i*} to frame {*i* + 1} can be derived as follows:

(1) $$\begin{array}{cc} T_{i}^{i-1} & =R_{X}(a_{i})D_{X}(a_{i})R_{Z}(\theta_{i})D_{Z}(d_{i})\\ & =\left[\begin{array}{cccc} \cos\theta_{i} & -\sin\theta_{i} & 0 & a_{i}\\ \sin\theta_{i}\cos\alpha_{i} & \cos\theta_{i}\cos\alpha_{i} & -\sin\alpha_{i} & -d_{i}\sin\alpha_{i}\\ \sin\theta_{i}\cos\alpha_{i} & \cos\theta_{i}\sin\alpha_{i} & \cos\alpha_{i} & d_{i}\cos\alpha_{i}\\ 0 & 0 & 0 & 1 \end{array}\right] \end{array}$$

where *α_i_*, *a_i_*, *θ_i_*, and *d_i_* are the standard D-H parameters describing the relative geometry between links (*i*−1) and *i*, namely the link twist, link length, joint rotation, and link offset, respectively. Accordingly, *R_X_*(*α_i_*) and *R_Z_*(*θ_i_*) denote the rotation matrices generated by *α_i_* and *θ_i_* about the X- and Z-axes, while *D_X_*(*a_i_*) and *D_Z_*(*d_i_*) represent the corresponding translations along the X- and Z-axes.

Based on Equation (1), the successive link-to-link transformations $T_{1}^{0},T_{2}^{1},T_{3}^{2},T_{4}^{3},T_{5}^{4}$ can be obtained. Their ordered product yields the overall homogeneous transformation from the base frame to the end-effector frame, denoted by $T_{5}^{0}$, as expressed in Equation (2):

(2) $$T_{5}^{0}=T_{1}^{0}T_{2}^{1}T_{3}^{2}T_{4}^{3}T_{5}^{4}=\left[\begin{array}{cccc} n_{x} & o_{x} & a_{x} & p_{x}\\ n_{y} & o_{y} & a_{y} & p_{y}\\ n_{z} & o_{z} & a_{z} & p_{z}\\ 0 & 0 & 0 & 1 \end{array}\right]=T(\theta_{1},\theta_{2},\theta_{3},\theta_{4},\theta_{5})=\left[\begin{array}{cc} R & P\\ 0 & 1 \end{array}\right]$$

In the end-effector frame, the orientation can be represented by three mutually orthogonal unit vectors *n*, *o*, and *a*, which define the tool’s local axes and satisfy the right-hand convention. Here, **a** is commonly interpreted as the approach direction of the end effector, **o** indicates the orientation (often associated with the tool’s transverse axis), and **n** completes the triad as the normal axis. The end-effector position is given by *p* = [*p_x_*, *p_y_*, *p_z_*]. Accordingly, the pose of the manipulator can be expressed as a homogeneous transformation *T* (*θ*_1_, *θ*_2_, *θ*_3_, *θ*_4_, *θ*_5_), obtained by chaining the link transformations between consecutive joint coordinate frames. In this formulation, $R\in {\mathbb{R}}^{3\times 3}$ denotes the rotation matrix mapping the end-effector frame to the base frame, while *p* denotes the translation (position) of the end-effector origin expressed in the base coordinate system.

To compute inverse kinematics for rehabilitation robotic arms, we first express the end-effector pose in the base coordinate system using a homogeneous transformation. Let the desired pose be:

(3) $$T_{d}=\left[\begin{array}{cc} R_{d} & P_{d}\\ 0 & 1 \end{array}\right]$$

where $R_{d}\in {\mathbb{R}}^{3\times 3}$ and $P_{d}\in {\mathbb{R}}^{3}$ denote the desired orientation and position, respectively.

For a given joint vector $\theta =[\theta_{1},\theta_{2},\theta_{3},\theta_{4},\theta_{5}]$, the forward kinematics yields the current pose:

(4) $$T(\theta )=\left[\begin{array}{cc} R(\theta ) & P(\theta )\\ 0 & 1 \end{array}\right]$$

The inverse kinematics problem seeks a feasible *θ* such that *T*(*θ*) matches *T_d_* as closely as possible.

To quantify the mismatch between *T*(*θ*) and *T_d_*, the pose error is decomposed into a translational component and a rotational component. The position error is defined as:

(5) $$e_{p}(\theta )=P(\theta )-P_{d}$$

The magnitude ${\left\|e_{p}(\theta )\right\|}_{2}$ measures the Euclidean distance between the current and desired end-effector positions.

For the orientation error, instead of using Euler-angle differences that may suffer from singularities, we adopt a rotation-matrix-based metric on *SO*(3). The relative rotation between the current and desired orientations is:

(6) $$R_{e}(\theta )=R_{d}^{T}R(\theta )$$

The corresponding geodesic rotation-angle error is:

(7) $$e_{R}(\theta )=\cos^{-1}(\frac{tr(R_{e}(\theta )-1)}{2})$$

where tr(⋅) denotes the matrix trace. Here, *e_R_*(*θ*) ∈ [0, π] is the minimum rotation angle required to align *R*(*θ*) with *R_d_*; smaller values indicate better orientation matching.

Safety requirements in rehabilitation robotics impose strict joint-range constraints. Therefore, each joint variable *θ*(*i*) must satisfy:

(8) $$\theta_{i}^{\min}\leq \theta_{i}\leq \theta_{i}^{\max},i=1,2,\cdots ,n$$

where $\theta {\left(i\right)}_{\min}$ and $\theta {\left(i\right)}_{\max}$ denote the lower and upper bounds of the *i*-th joint, respectively. These constraints prevent infeasible or unsafe motions during training.

With the above definitions, the inverse kinematics problem is reformulated as a constrained optimization problem under joint limits:

(9) $$\begin{array}{l} \underset{\theta }{\min}f(\theta )\\ s.t.\;\begin{array}{c} \theta_{i}^{\min} \end{array}\leq \theta_{i}\leq \theta_{i}^{\max},i=1,\cdots ,n \end{array}$$

where the objective function is defined as a normalized weighted sum of the position error and orientation error:

(10) $$\begin{array}{l} f(\theta )=w_{p}{\left\|{\tilde{e}}_{p}(\theta )\right\|}_{2}+w_{R}{\tilde{e}}_{R}(\theta ),\\ w_{p}+w_{R}=1\\ {\tilde{e}}_{p}(\theta )=\frac{e_{p}(\theta )}{L_{ref}},\;{\tilde{e}}_{R}(\theta )=\frac{e_{R}(\theta )}{\pi } \end{array}$$

where *L_ref_* is a characteristic length used to normalize the translational error, and π denotes the maximum possible geodesic rotation angle on *SO*(3). In this way, both error terms become dimensionless and comparable in magnitude. In this study, *L_ref_* is chosen as the characteristic arm length of the rehabilitation robot. Unless otherwise specified, *w_p_* = *w_R_* = 0.5 is adopted to provide a balanced treatment of translational and rotational accuracy.

## 3. Biomimetic Beaver-Inspired Optimization for Inverse Kinematics of Rehabilitation Robotic Arms

The BBO is a bio-inspired population-based metaheuristic that models the collective survival strategies observed in beavers during habitat construction and resource management [18]. In nature, beavers exhibit a mixture of cooperative building, information sharing, and adaptive exploration when searching for suitable locations and materials to construct dams and lodges. Such behaviors naturally reflect two complementary search patterns in optimization: global exploration, where individuals probe different regions to maintain diversity and avoid premature convergence, and local exploitation, where promising solutions are refined by learning from high-quality experiences. Motivated by this principle, BBO represents each candidate solution as an individual “beaver” and updates the population through an iteration-dependent transition mechanism that gradually shifts the search from exploration to exploitation. In the exploration stage, the population can be divided into elite “architects” and non-elite “explorers”, where architects emphasize structured information exchange among good solutions, while explorers perform guided learning combined with stochastic perturbations to discover unseen regions. In the exploitation stage, individuals refine their states by jointly learning from randomly selected peers and the current global best solution, thereby accelerating convergence. Owing to its simple structure, derivative-free nature, and strong adaptability to nonlinear constrained problems, BBO provides a convenient optimization backbone for inverse kinematics, where the objective is typically nonconvex and admits multiple feasible joint configurations under joint-limit constraints.

### 3.1. Beaver Behavior Optimizer-Based Inverse Kinematics

BBO is a population-based metaheuristic. Each candidate solution (one “beaver”) is a joint vector *θ_i_*(*t*) at iteration *t*:

(11) $$\theta_{i}(k)=[\theta_{i,1}(t),\cdots ,\theta_{i,D}(t)],\;t=1,\cdots ,N$$

where *N* is the population size, *T* denotes the maximum number of iterations, and *D* is the number of joints (decision variables).

Each joint is initialized uniformly within limits:

(12) $$\theta_{i,j}=\theta_{j}^{l}+(\theta_{j}^{u}-\theta_{j}^{l})\cdot rand,\;i=1,\cdots ,N;\;j=1,\cdots ,D$$

where *rand*∼U(0,1). *θ*, *θ_j_* are joint vector and its *j*-th component (radians), respectively. $\theta_{j}^{u},\theta_{j}^{l}$ represent lower and upper joint limits, respectively. Compute fitness $f(\theta_{i,j})$ using Equation (9), and store the best solution:

(13) $$\theta_{best}(t)=\arg\underset{\theta_{i}(t)}{\min}f(\theta_{i}(t))$$

BBO uses a smooth increasing factor to shift from global exploration to local exploitation:

(14) $$E(t)=\sin(\frac{\pi t}{2T}),\;t=1,\cdots ,T$$

Draw r∼U(0,1). If *r* ≤ E(*t*), execute exploitation; otherwise, execute exploration.

In exploitation, each beaver learns from a randomly selected peer *k* ≠ *i* and from the global best:

(15) $$\theta_{i,j}(t+1)=\theta_{i,j}(t)+r_{1}(\theta_{k,j}(t)-\theta_{i,j}(t))+r_{2}(\theta_{best,j}(t)-\theta_{i,j}(t))$$

where *r*_1_, *r*_2_∼U(0,1). This update encourages rapid improvement by contracting the search around promising regions.

It should be emphasized that this peer-and-best learning pattern is an inherent part of the original BBO dam-maintenance exploitation mechanism, and it is presented here as a baseline component rather than a newly introduced operator.

In exploration, the population is sorted by fitness. The top fraction (e.g., 25%) forms an elite set of architects A, and the rest are explorers. 

Architect update (elite mutual learning). For an architect i∈A, randomly pick another architect k∈A:

(16) $$\theta_{i,j}(t+1)=\theta_{i,j}(t)+\prod (r_{3}<0.5)+r_{4}(\theta_{k,j}(t)-\theta_{i,j}(t))$$

where *r*_3_, *r*_4_∼U(0,1). ∏(⋅) is the indicator function.

Explorer update (learn from elites + stochastic probing). For an explorer i∉A, pick an architect k∈A and apply:

(17) $$\theta_{i,j}(t+1)=\theta_{i,j}(t)+\prod (r_{5}<0.5)\cdot r_{6}(\theta_{k,j}(t)-\theta_{i,j}(t))+\sigma_{j}(t)\cdot N(0,1)$$

where *r*_5_, *r*_6_∼U(0,1). N(0,1) is standard Gaussian noise, and the noise amplitude decays with iterations:

(18) $$\sigma_{j}(t)=\cos(\frac{\pi t}{2T})\cdot \frac{\theta_{j}^{u}-\theta_{j}^{l}}{\eta }$$

where *η* is a scaling factor that controls the magnitude of the Gaussian perturbation.

Thus, early iterations explore broadly; later iterations reduce randomness to preserve convergence.

After updating, each joint is clamped to its feasible interval:

(19) $$\theta_{i,j}(t+1)=\min(\max(\theta_{i,j}(t+1),\;\theta_{j}^{l}),\;\theta_{j}^{u})$$

Then evaluate the new fitness $f(\theta_{i,j}(t+1))$. A common acceptance rule is greedy:

(20) $$\theta_{i}(t+1)=\left\{\begin{array}{l} \theta_{i}(t+1),\;f(\theta_{i}(t+1))<f(\theta_{i}(t))\\ \theta_{i}(t),\;otherwise \end{array}\right.$$

and update $\theta_{best}(t)$ according to Equation (13).

After *T* iterations, BBO outputs the inverse kinematics solution:

(21) $$\theta^{*}=\theta_{best}(T),\;f^{*}=f(\theta^{*})$$

### 3.2. Improved Beaver Behavior Optimizer for Inverse Kinematics

To improve the terminal accuracy and numerical stability of BBO for highly nonlinear inverse kinematics, the Improved Beaver Behavior Optimizer (IBBO) is adopted as the search engine. The baseline BBO exploration framework described in Section 3.1 is preserved, including the architect–explorer role division and the associated update rules. Four targeted mechanisms are incorporated to enhance precision-oriented convergence: (1) rollback-consistent elitist replacement to preserve state–fitness consistency; (2) momentum-guided exploitation to promote smoother late-stage refinement; (3) late-stage coordinate-wise refinement for low-cost tail optimization; and (4) an adaptive architect ratio during exploration to regulate information flow and maintain population diversity across iterations. Under the same joint-limit constraints, these mechanisms are integrated into the iterative optimization of joint vectors *θ_i_*(*t*), resulting in more reliable convergence and reduced terminal pose mismatch.

(1) Elitist replacement with rollback consistency

For the *i*-th individual, a trial candidate ${\theta^{\prime }}_{i}(t)$ is generated by the update rule (either exploration or exploitation), and its fitness is computed as ${f^{\prime }}_{i}(t)=f({\theta^{\prime }}_{i}(t))$. IBBO adopts a strict elitist acceptance with rollback to ensure that the stored state “position–fitness” is always consistent:

(22) $$\theta_{i}(t+1)=\left\{\begin{array}{l} {\theta^{\prime }}_{i}(t),\;{f^{\prime }}_{i}(t)<f(\theta_{i}(t))\\ \theta_{i}(t),\;otherwise \end{array}\right.,\;f(\theta_{i}(t+1))=\left\{\begin{array}{l} {f^{\prime }}_{i}(t),\;{f^{\prime }}_{i}(t)<f(\theta_{i}(t))\\ f(\theta_{i}(t)),\;otherwise \end{array}\right.$$

Then the global best is updated accordingly:

(23) $$\theta_{best}(t+1)=\arg\underset{i\in \{1,\cdots ,N\}}{\min}f(\theta_{i}(t+1))$$

where ${\theta^{\prime }}_{i}(t)$ denotes the trial joint vector generated at iteration *t*, ${f^{\prime }}_{i}(t)$ is the corresponding fitness value, and the rollback rule restores *θ_i_*(*t*) whenever no improvement is achieved, thereby preventing hidden “position updated but fitness not updated” inconsistencies.

(2) Momentum-exploitation update

The exploitation update keeps the original BBO dam-maintenance learning structure unchanged; the proposed enhancement targets late-stage stability and solution polishing through a momentum-style information carryover within the same BBO exploitation framework. In the exploitation stage, IBBO introduces a momentum memory $v_{i}(t)=[v_{i,1}(t),\cdots ,v_{i,D}(t)]$ to guide late-stage refinement. Select a random peer *k* ≠ *i*. The momentum update is:

(24) $$v_{i}(t+1)=w(t)v_{i}(t)+c_{1}(t)r_{1}⊙(\theta_{k}(t)-\theta_{i}(t))+c_{2}(t)r_{2}⊙(\theta_{best}(t)-\theta_{i}(t))$$

where *w*(*t*) is the inertia weight, *c*_1_(*t*) and *c*_2_(*t*) are learning coefficients, *r*_1_, *r*_2_ ∈ [0, 1] are element-wise random vectors, ⊙ denotes the Hadamard product, *v_i_*(*t*) is momentum vector of the *i*-th individual.

The trial position is generated by:

(25) $${\theta^{\prime }}_{i}(t)=\theta_{i}(t)+v_{i}(t+1)+\varepsilon (t)$$

where *ε*(t) is the perturbation, a small Gaussian noise with a decaying amplitude:

(26) $$\varepsilon (t)\sim N(0,\;\sigma^{2}(t)I),\;\sigma (t)=\alpha (1-\frac{t}{T})(u-1)$$

where σ(t) is the iteration-dependent standard deviation, which gradually decays as the iteration index *t* increases.

(3) Late-stage coordinate refinement (memetic local search)

To further reduce the residual inverse kinematics error in the tail stage, IBBO performs a lightweight coordinate refinement on the current best solution *θ*_best_(*t*) when *t* > 0.7*T*. Every *K* iterations, for the *j*-th coordinate, a rapidly decaying step size is defined as:

(27) $$\Delta_{j}(t)=\Delta_{0}{\left(1-\frac{t}{T}\right)}^{2}(\theta_{j}^{u}-\theta_{j}^{l})$$

where $\Delta_{j}(t)$ is coordinate refinement step size, $\Delta_{0}$ denotes initial refinement scale, *T* represents the maximum number of iterations.

Two trial candidates are constructed:

(28) $$\theta^{+}=\theta_{best}(t)+\Delta_{j}(t)e_{j},\;\theta^{-}=\theta_{best}(t)-\Delta_{j}(t)e_{j}$$

where *e_j_* is the *j*-th standard basis vector. After clamping *θ*^+^ and *θ*^−^ to joint limits, the best solution is refined as:

(29) $$\theta_{best}(t)\leftarrow \arg\min\{f(\theta_{best}(t)),\;f(\theta^{+}),\;f(\theta^{-})\}$$

(4) Iteration-dependent architect ratio schedule in exploration stage

To avoid using a fixed architect proportion (e.g., 25%) throughout the search, IBBO introduces an iteration-dependent architect ratio in the exploration stage. This adaptive design aims to allocate more architects in early iterations to form effective guidance channels and gradually reduce the architect proportion to preserve diversity and prevent premature convergence in the later stage.

Specifically, the architect ratio is scheduled as:

(30) $$\rho_{A}(t)=\rho_{\max}-(\rho_{\max}-\rho_{\min})\frac{t}{T},\;t=1,\cdots ,T$$

where *ρ_A_*(*t*) is the architect ratio at iteration *t*. *ρ*_max_ and *ρ*_min_ are upper and lower bounds of architect ratio, respectively.

The linear schedule is adopted as a simple and reproducible annealing strategy to gradually relax elite dominance and preserve diversity toward the end of the search. Compared with feedback-driven schedules, this design introduces no extra diversity estimator or tuning parameters, which improves stability.

The number of architects at iteration *t* is determined by:

(31) $$N_{A}(t)=\max\{2,\;\left\lfloor \rho_{A}(t)N\right\rfloor \}$$

where ⌊⋅⌋ denotes the floor operator.

After sorting individuals by fitness (ascending order), the architect set A(t) is constructed as the indices of the top *N_A_*(*t*) individuals:

(32) $$A(t)=\{i|i\in Top-N_{A}(t)\;\begin{array}{cc} accrding & to \end{array}f(\theta_{i}(t))\}$$

Then, the architect update and explorer update in the exploration stage follow the same forms as Equations (16) and (17) in Section 3.1, except that the architect set A is replaced by A(t), and the architect count is no longer fixed.

Algorithm 1 presents the detailed pseudocode of the proposed IBBO. The algorithmic flow is organized in a step-by-step manner, including population initialization, fitness evaluation, iterative position updating, and termination checking. Each major operator and update rule is explicitly specified to ensure reproducibility and to clarify how IBBO balances exploration and exploitation across iterations.
**Algorithm 1 IBBO****Input:** objective function *f*(⋅), population size *N*, max iteration *T*, dimension *D*, joint bounds *θ^l^*, *θ^u^***/--Note: Initialization--/**1.**Initialize:** population *θ_i_*←*θ^l^* + (*θ^u^* − *θ^l^*)⊙ rand (1,D) for *i* = 1, …, *N*.2.Evaluate fitness *f_i_*←*f*(*θ_i_*) for *i* = 1, …, *N*.3.Set global best (*θ*_∗_,*f*_∗_)←argmin*_i_f_i_*4.Initialize momentum *v_i_*←0 for all *i*.5.Set stagnation counters: *stall*←0, *lastBest*←*f*_∗_.6.**for** *t =* 1 to *T*7.   Compute phase factor *E*←sin (π/2)* t*/*T*).8.   Compute architect ratio *ρ_A_*(*t*)←*ρ*_max_ − (*ρ*_max_ − *ρ*_min_)·*t*/*T*9.   *N_A_*(*t*)←max(2, *round*(*ρ_A_*(*t*)*N*)).10.   Sort individuals by fitness (ascending) and set architects *A* as the best *N_A_*(*t*) indices.11.   Update momentum parameters *w*(*t*), *c*_1_(*t*), *c*_2_(*t*)12.   **for** *i* = 1 to *N*13.      Store rollback state: *θ_i_*←*θ_i_*, *f_i_*←*f_i_.*14.      **if** rand < *E* **then** (Exploitation with momentum)15.          Randomly choose *k* ≠ *i*, draw *r*_1_, *r*_2_ ∈ [0, 1]16.          Update velocity *v_i_*←*w*(*t*)*v_i_* + *c*_1_(*t*)*r*_1_⊙(*θ_k_* − *θ_i_*) + *c*_2_(*t*)*r*_2_⊙(*θ*_∗_ − *θ_i_*).17.          Generate candidate *θ*′*_i_*←*_i_* + *v_i_* + *ε*(*t*),18.          where *ε*(*t*)∼*N*(0, *σ*^2^(*t*)**I**) and *σ*(*t*) = *α*(1 − *t*/*T*)(*θ^u^* − *θ^l^*).19.      **else** (Exploration with roles)20.           ***if*** *i*∈A* **then*** (Architect update)21.               *Choose k* ∈ A,* k* ≠ *i.* For each dimension *j*:22.               *θ*′*_i_*_,*j*_←*θ_i_*_,*j*_ + Π(*r*_3_ < 0.5)*r*_4_(*θ_k_*_,*j*_ − *θ_i_*_,*j*_), Π(⋅) ∈ {0,1}23.           ***else*** (***Explorer*** update)24.               Choose *k* ∈ A. For each dimension *j*:25.               with prob. 0.5: *θ*′*_i_*_,*j*_←*θ_i_*_,*j*_ + *r*_4_(*θ_k_*_,*j*_ − *θ_i_*_,*j*_);26.               otherwise perturb:27.               *θ′_i_*_,*j*_←*θ_i_*_,*j*_ + cos(((π/2)*t*/*T*))($\theta_{j}^{u}$ − $\theta_{j}^{l}$)N(0,1)/*η*28.
           ***end if***
29.
      **end if**
30.      Clamp bounds: *θ*′*_i_*←min(max(*θ*′*_i_*,*θ^l^*),*θ^u^*).31.      Evaluate candidate fitness *f_i_*′←*f*(*θ*′*_i_*).32.      **if** *f_i_*′ < *f_i_* **then** *θ_i_*←*θ_i_*′, *f_i_*←*f_i_*′33.      **else** rollback *θ_i_*←*θ_i_*, *f_i_*←*f_i_*.34.
      **end if**
35.      **if** *f_i_* < *f_∗_* **then** (*θ*_∗_,*f*_∗_)←(*θ_i_*,*f_i_*).36.
      **end if**
37.
   **end for**
38.   **if** *t* > 0.7 *T* and mod(*t*,*K*) = 0 **then** perform coordinate refinement on *θ*_∗_:39.      Try *θ*_∗_^+^ = *θ*^∗^ + Δ*_j_*(*t*)*e_j_*, *θ*_∗_^−^ = *θ*_∗_ − Δ*_j_*(*t*)*e_j_*,40.      where Δ*j*(*t*) = Δ_0_(1 − *t*/*T*)^2^($\theta_{j}^{u}$ − $\theta_{j}^{l}$), keep the best.41.
   **end if**
42.   Record convergence *f*_∗_(*t*)←*f*_∗_.43.**end for**44.Return *θ*_∗_, *f*_∗_**Output:** best joint solution *θ*_∗_, best fitness *f*_∗._

This section establishes a complete optimization-based framework for solving the inverse kinematics of rehabilitation robots. The inverse kinematics task is first formulated as a continuous constrained optimization problem with joint-limit bounds, where the pose discrepancy is defined as the fitness to be minimized, and a BBO-driven population search is adopted to iteratively evolve candidate joint vectors from global exploration to local exploitation. On this basis, the IBBO is incorporated to further strengthen precision-oriented convergence without changing the baseline BBO exploration structure. Specifically, rollback-consistent elitist replacement is introduced to maintain strict state–fitness consistency, a momentum-guided exploitation update is embedded to enable a smoother transition from exploration to fine refinement and improve late-stage accuracy, and a late-stage coordinate refinement is applied to achieve low-cost tail optimization. In addition, an adaptive architect ratio is employed in the exploration stage to dynamically balance guidance intensity and population diversity over iterations. Collectively, these mechanisms enhance convergence reliability and terminal accuracy under identical joint constraints and provide a unified and reproducible procedure to obtain the optimal joint solution *θ*^∗^ for subsequent experiments and practical deployment.

### 3.3. Convergence and Complexity Analysis of IBBO

#### 3.3.1. Convergence Discussion

IBBO is a stochastic population-based metaheuristic. Let the population at iteration *t* be $\Theta (t)={\left\{\theta_{i}(t)\right\}}_{i=1}^{N}$, and let *f*(*θ*) denote the objective function. The algorithm evolves Θ(*t*) through phase-controlled exploitation and exploration updates, combined with feasibility preservation, elitist acceptance, and late-stage local refinement.

Boundedness and feasibility preservation. All candidate joint vectors are constrained within the feasible domain $\Omega =[\theta^{l},\;\theta^{u}]$ by the bound-clamping operation after each update. Therefore, the search trajectory of every individual remains bounded and feasible throughout the evolution. This boundedness prevents numerical divergence and ensures that the optimization process is well-defined over a compact domain.

Monotonic best-so-far property induced by rollback-consistent elitism. IBBO adopts a strict elitist acceptance with rollback. For each individual, the pre-update state is stored, and the updated candidate is accepted only if it yields a better fitness value; otherwise, the individual is restored to its previous state. Consequently, the best-so-far fitness value *f*_best_(*t*) is monotonically non-increasing for minimization problems, because any accepted update cannot worsen the current best, and any rejected update is discarded via rollback. This property guarantees that the algorithm never loses the best solution found so far and provides a stable descending sequence of best-so-far objective values.

Stochastic reachability and global exploration capability. IBBO contains multiple sources of randomness, including phase switching between exploitation and exploration, random selection of peers/architects, and stochastic perturbations in the exploration updates. In particular, the exploration stage introduces continuous random perturbations, which yield a nonzero probability of generating candidate solutions in different neighborhoods within the feasible domain. Under mild regularity assumptions, these stochastic operators imply that IBBO can repeatedly sample the feasible space with nonzero probability while preserving the best solution via elitist rollback. This constitutes the standard sufficient condition used in the metaheuristic literature for global convergence in probability, meaning that the probability of visiting the global optimum increases as the number of iterations grows.

Late-stage refinement and enhancement of terminal accuracy. In addition to population evolution, IBBO activates a coordinate-wise local refinement for the best solution in the late stage of the run. This refinement probes the current best along coordinate directions with a step size that shrinks over iterations, and only improving moves are retained. Once the population enters a high-quality basin of attraction, such a shrinking-step local search increases the likelihood of further decreasing the objective and strengthens tail-end exploitation, which is particularly important for inverse kinematics tasks that demand high terminal pose accuracy.

The convergence behavior of IBBO is supported by three complementary properties: bounded feasible search enforced by clamping, monotonic non-increasing best-so-far fitness ensured by rollback-consistent elitism, and nonzero-probability exploration due to stochastic phase-controlled updates. The additional late-stage coordinate refinement further improves local convergence behavior and final solution polishing near high-quality feasible regions.

#### 3.3.2. Computational Complexity

Let *N* be the population size, *T* the maximum number of iterations, and *D* the decision dimension (number of joints). Denote by *C_f_* the computational cost of one fitness evaluation, which typically includes forward kinematics computation and pose-error evaluation in the inverse kinematics setting.

In each iteration, generating candidate solutions and performing element-wise updates, including exploitation/exploration updates and bound clamping, require O(ND) time. Identifying architects requires sorting the population by fitness, which costs O(NlogN) per iteration. Fitness evaluation dominates the runtime and costs $O(NC_{f})$ per iteration because each candidate solution must be evaluated once.

Therefore, the overall complexity of the main evolutionary loop is

(33) $$O(T(NC_{f}+ND+N\log N))$$

Cost of late-stage coordinate refinement. The coordinate-wise refinement is executed intermittently in the late stage. Each invocation probes the best solution along coordinate directions and requires $O(DC_{f})$ time (up to a small constant factor depending on the number of trial moves per coordinate). If the refinement is triggered every *K* iterations in the final portion of the run, the additional cost is approximately:

(34) $$O(\frac{T}{K}DC_{f})$$

which is typically negligible compared with $O(TNC_{f})$ when *N* is moderate or large.

Memory complexity. IBBO stores the population Θ(*t*) and auxiliary vectors such as momentum and temporary candidates. The dominant memory usage is O(ND), while additional scalars (best fitness, counters, and scheduling parameters) contribute negligible overhead.

## 4. Experimental Validation

### 4.1. Benchmark Function Experiments

This section reports numerical experiments on benchmark functions to evaluate the convergence behavior and overall optimization performance of the proposed IBBO. To ensure a fair and statistically reliable comparison, IBBO is evaluated against the original BBO, PSO [19], the puma optimizer algorithm (PO) [20], and the whale optimization algorithm (WOA) [21], using identical parameter settings and termination criteria. Each algorithm is independently run 30 times on each benchmark function. The mean (Ave) and standard deviation (Std) of the best objective values obtained are reported, where AVG reflects overall solution quality and STD quantifies robustness and run-to-run stability. In addition, algorithms are ranked on each test function according to their performance (lower ranks indicate better performance). The ranks are then aggregated across all functions to produce an overall ranking, providing a comprehensive assessment of IBBO’s competitiveness relative to the baseline methods.

**Figure 2:**
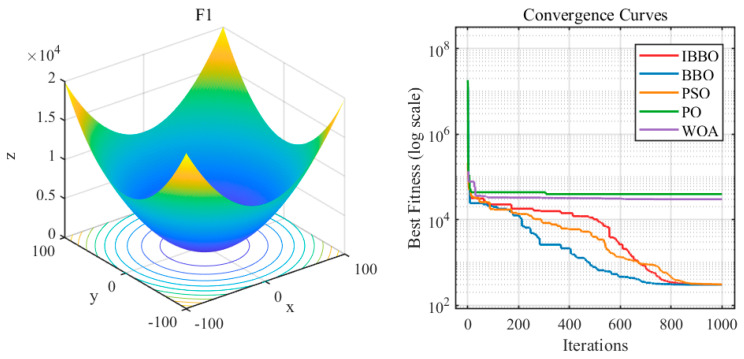

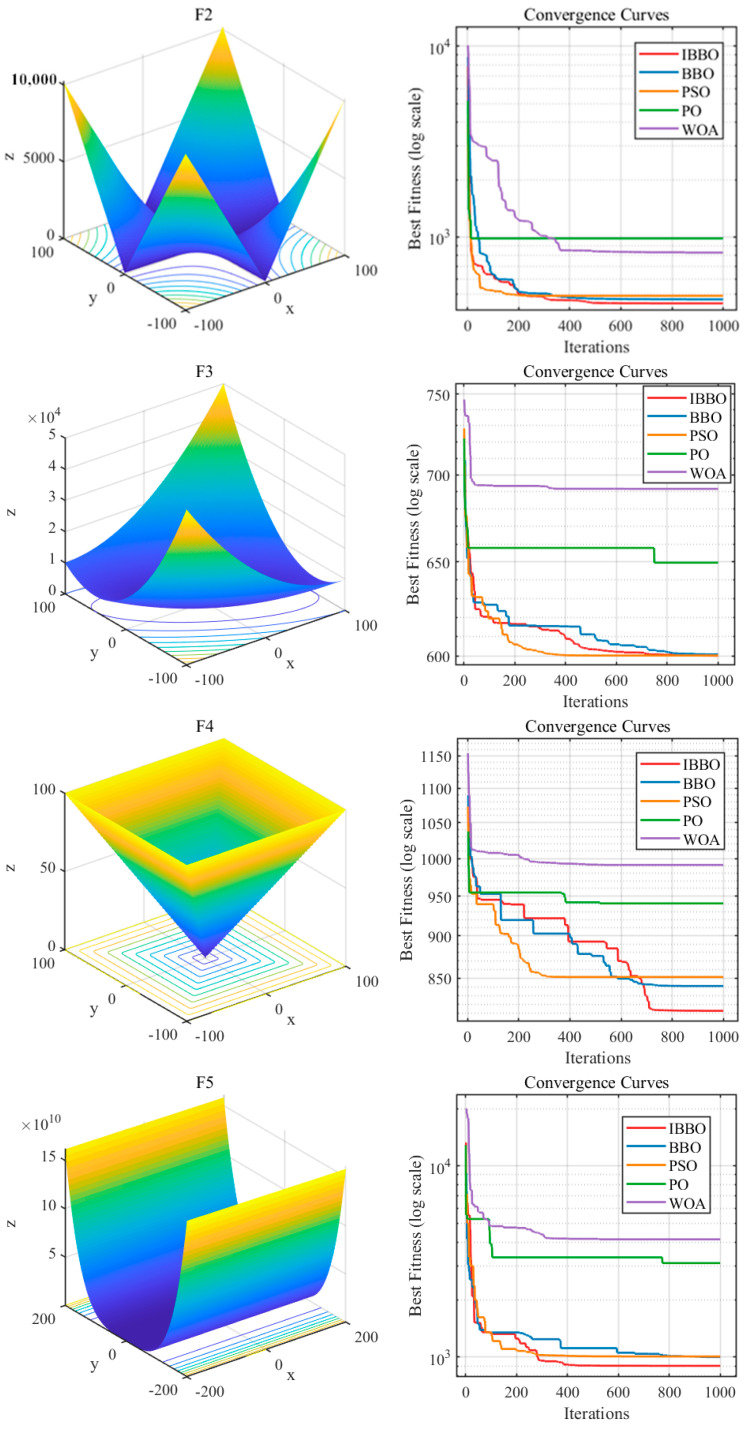

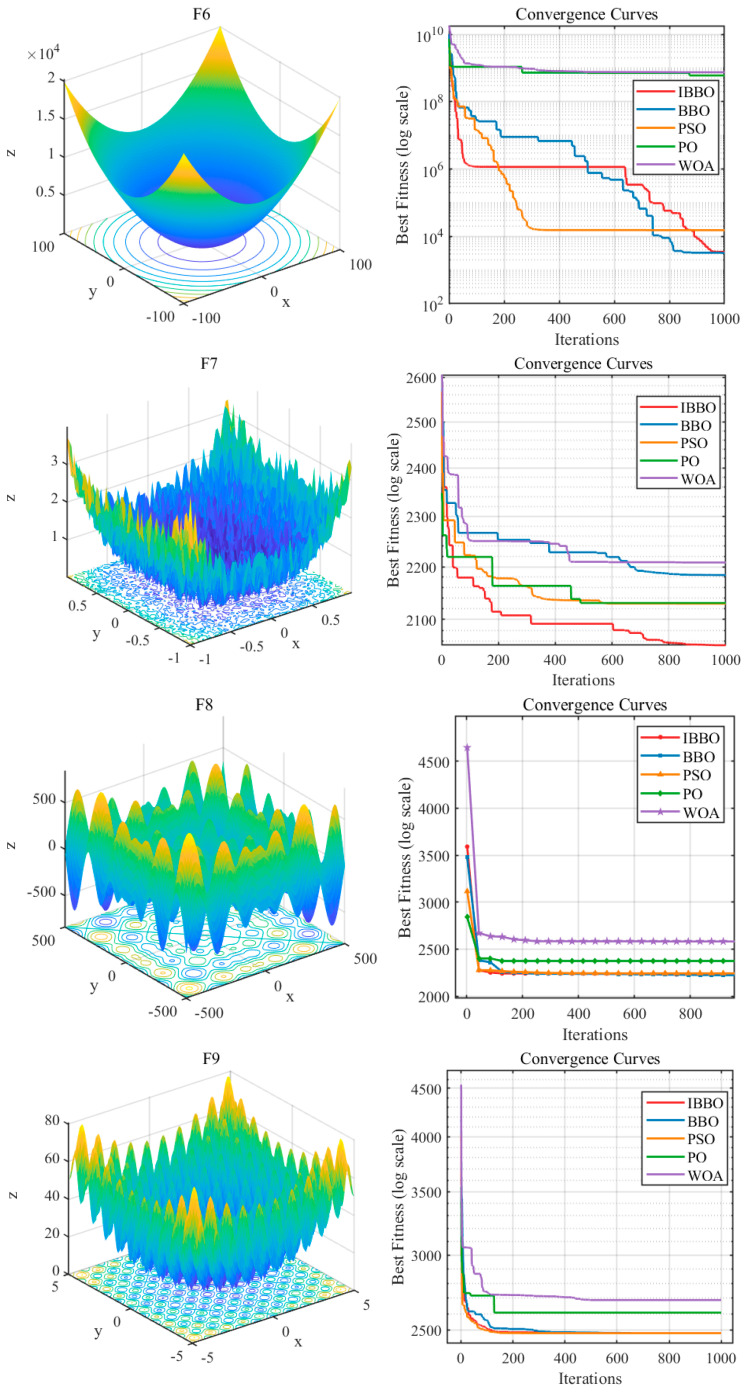

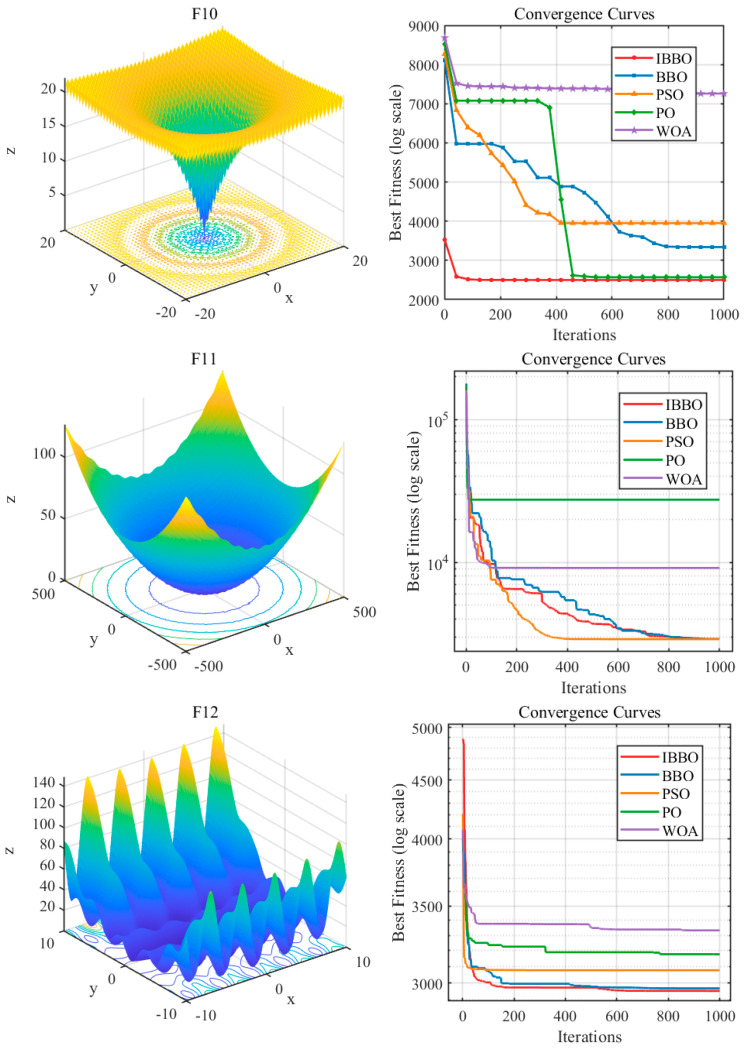
Progression of best fitness over iterations on the CEC 2022 benchmark suite.

A comprehensive performance evaluation of IBBO was conducted against representative competitors on the CEC2022 benchmark suite under 20-dimensional scenarios. Figure 2 illustrates the convergence behaviors by plotting the progression of the best fitness value over iterations for each test function, enabling a direct comparison of convergence speed and final solution quality across algorithms. Table 2 reports the quantitative results in terms of the Ave and Std over 30 independent runs, reflecting both optimization accuracy and robustness. In addition, Table 3 summarizes the statistical analyses, including F-ranking, the Wilcoxon signed-rank test, and the corresponding *p*-values (predominantly *p* < 0.05), to rigorously assess whether IBBO achieves statistically significant improvements over competing methods on each function.

Figure 2 illustrates the convergence profiles of IBBO and four competing algorithms on the CEC2022 benchmark suite by plotting the best-so-far fitness over 1000 iterations (the fitness axis is shown on a logarithmic scale). Overall, IBBO demonstrates a more rapid reduction in fitness during the early iterations for most test functions, suggesting an effective global exploration ability that enables the search to move quickly away from poor regions of the landscape. In the subsequent iterations, the IBBO curve typically continues to decrease smoothly with limited oscillations or abrupt rebounds, indicating stable exploitation and reliable refinement. For several representative functions (e.g., F4, F7–F10, and F12), IBBO not only converges faster but also attains lower terminal fitness than the compared methods, implying superior solution quality and a diminished risk of premature convergence. In contrast, PO and WOA often exhibit early stagnation, characterized by long plateaus at comparatively higher fitness values, which suggests inadequate local improvement once the search becomes trapped in suboptimal basins. Although some competitors remain competitive on a few functions (e.g., F3 and F11) in the later stage, IBBO consistently maintains a close or leading performance across the suite, underscoring its favorable trade-off between exploration and exploitation in the 20-dimensional setting.

Table 2 reports the quantitative performance of IBBO and the competing algorithms on the CEC2022 benchmark suite in the 20-dimensional setting, where the Ave and Std are computed over 30 independent runs. Overall, IBBO achieves the lowest mean objective value on 11 of the 12 functions and, concurrently, yields the smallest Std on 11 of the 12 functions, indicating consistently superior accuracy together with strong robustness. Notably, IBBO demonstrates clear advantages on challenging problems with large dynamic ranges, such as F1 and F6, where it attains objective values that are orders of magnitude lower than those produced by PSO, PO, and WOA, while also exhibiting substantially reduced run-to-run variability. For another subset of functions (e.g., F4–F5 and F7–F10), IBBO not only converges to lower final fitness levels than the competing methods but also maintains markedly smaller dispersion, suggesting reliable convergence behavior and a reduced propensity for premature convergence in suboptimal basins. The only exception is F11, on which PSO attains a marginally lower mean; however, IBBO remains highly competitive with a negligible gap and preserves stable performance. Taken together, the results in Table 2 substantiate the effectiveness of IBBO in achieving a favorable trade-off between exploration and exploitation and highlight its strong performance consistency across diverse CEC2022 function categories under 20-dimensional scenarios.

Table 3 provides a statistical verification of the comparative results by reporting the per-function F-ranking alongside pairwise Wilcoxon signed-rank tests with IBBO as the reference method under the CEC2022 20-dimensional setting. IBBO achieves the top rank (F-ranking = 1) on 11 of the 12 functions, which is consistent with its overall superiority across the benchmark suite. More importantly, for nearly all functions, PSO, PO, WOA, and BBO are annotated with Wilcoxon “(+)” and associated with *p* < 0.05, indicating that their performance is significantly inferior to IBBO and that IBBO’s improvements are unlikely to be explained by random variability across independent runs. This statistical evidence is particularly compelling in the comparison with the baseline BBO: although BBO frequently attains the second-best rank, the Wilcoxon results with *p* < 0.05 confirm that IBBO still delivers statistically significant gains on most functions. The only exception is F11, where PSO attains a better rank and exhibits a Wilcoxon “(−)” outcome (*p* < 0.05), implying a statistically significant advantage over IBBO for this specific case. Taken together, the ranking outcomes and significance tests substantiate the robustness and reliability of IBBO’s performance advantages across diverse CEC2022 problem categories in the 20-dimensional scenario.

### 4.2. Robot Inverse Kinematics Experiments

#### 4.2.1. Sensitivity Analysis of Weighting Coefficients

Before conducting the formal inverse kinematics comparison experiments, a sensitivity analysis of the weighting coefficients was carried out to determine a suitable trade-off between translational and rotational accuracy in the normalized objective function. As discussed in Section 2, the inverse kinematics objective is formulated as a weighted combination of the normalized position error and normalized orientation error, where the coefficients *w_p_* and *w_R_* control the relative emphasis on translation and rotation, respectively, under the constraint *w_p_* + *w_R_* = 1. Since different weight settings may bias the optimizer toward different solution characteristics, it is necessary to examine their influence before selecting the final configuration for subsequent experiments.

To this end, five representative weight settings were tested, namely (*w_p_*,*w_R_*) = (0.1,0.9), (0.3,0.7), (0.5,0.5), (0.7,0.3), and (0.9,0.1). For each setting, IBBO was independently run 20 times on the same representative inverse kinematics task, and the mean position error, mean angular error, and convergence behavior were recorded. It should be noted that the Frobenius-norm pose error was retained only as an auxiliary evaluation metric and was not included in the optimization objective itself.

**Figure 3:**
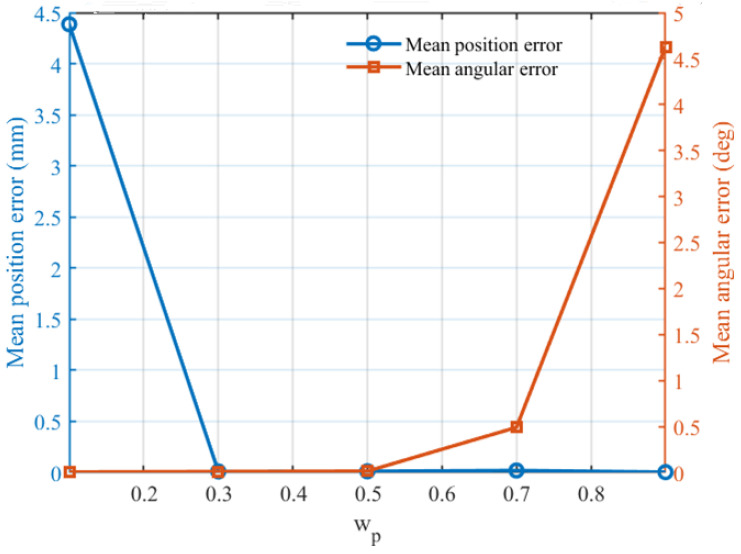
Sensitivity of translational and rotational accuracy to weight settings.

Figure 3 illustrates the sensitivity of translational and rotational accuracy to different weight settings. A clear trade-off can be observed. When the rotational term is overly emphasized, i.e., (*w_p_*,*w_R_*) = (0.1,0.9), the mean angular error remains very small, but the mean position error increases sharply, indicating that excessive preference for orientation matching may significantly degrade translational accuracy. Conversely, when the position term dominates, i.e., (*w_p_*,*w_R_*) = (0.9,0.1), the mean position error remains small, whereas the angular error rises substantially, showing that excessive emphasis on translation can impair orientation alignment. These results confirm that the two coefficients indeed regulate the balance between the two pose components and that extreme settings are not suitable for achieving overall inverse kinematics accuracy. More importantly, the intermediate settings (0.3,0.7), (0.5,0.5), and (0.7,0.3) all lead to much smaller errors than the two extreme cases, indicating that a moderate weighting strategy is preferable. Among them, (0.3,0.7) and (0.5,0.5) exhibit particularly favorable performance, with both translational and rotational errors remaining at consistently low levels. This suggests that the normalized objective formulation is effective and that the proposed solver is not overly sensitive to moderate weight variations around the balanced region.

**Figure 4:**
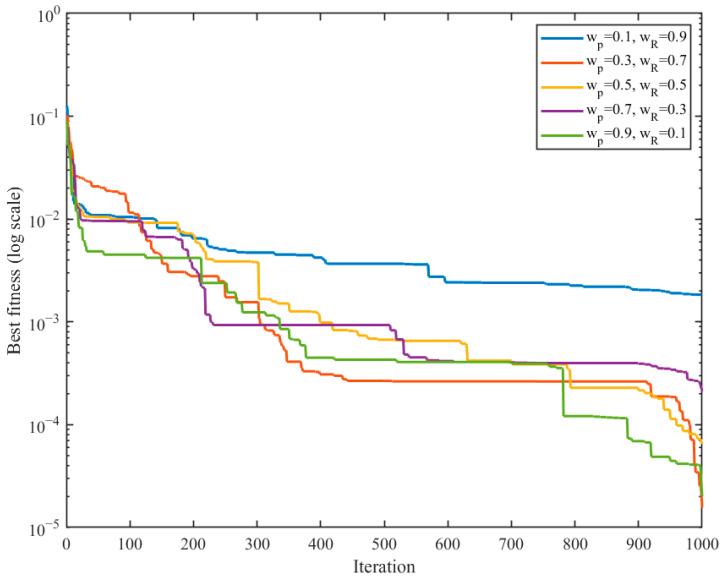
Convergence curves under different weight settings.

Figure 4 further compares the convergence curves under different weight settings. The setting (0.1,0.9) converges to a relatively high fitness plateau and shows limited late-stage refinement, implying that overemphasizing orientation is detrimental to the overall optimization objective. By contrast, the intermediate settings exhibit smoother and deeper convergence. In particular, (0.3,0.7) achieves the lowest terminal fitness and maintains a stable descending trend throughout the optimization process, indicating a favorable balance between early exploration and late-stage exploitation. Although (0.9,0.1) also reaches a low final fitness in the very late stage, its associated angular error in Figure 3 is clearly larger, which makes it less suitable for rehabilitation-oriented inverse kinematics tasks that require simultaneous translational and rotational consistency.

Considering both the accuracy trade-off in Figure 3 and the convergence quality in Figure 4, the weight setting (*w_p_*,*w_R_*) = (0.3,0.7) was selected for the subsequent inverse kinematics experiments. This setting provides low translational and rotational errors simultaneously while also yielding the most desirable convergence behavior among the tested candidates. Therefore, it was adopted as the default coefficient configuration in the following subsection.

#### 4.2.2. Inverse Kinematics Performance Evaluation

In this subsection, robot inverse kinematics is solved using the proposed IBBO algorithm, while the original BBO is adopted as a baseline for comparison. This choice is motivated by the benchmark-function evaluation in the previous section, which has already demonstrated the effectiveness and robustness of IBBO. The experiments are conducted on the rehabilitation mechanism under study. The robot is initialized at a given joint configuration *θ*_0_, where *θ*_0_ denotes the vector of initial joint angles. A target end-effector pose *p* is then selected (including both position and orientation). For each algorithm, an inverse kinematics solution is obtained and subsequently substituted into the forward kinematics model to compute the corresponding end-effector homogeneous transformation matrix. The predicted pose is compared with the desired target to quantify the inverse kinematics accuracy. Specifically, three evaluation metrics are adopted: the Frobenius-norm error of the end-effector pose transformation matrix, which quantifies the overall deviation between the predicted transformation and the target transformation; the position error, computed from the difference between the corresponding translational components; and the orientation error, obtained from the rotational components of the two transformations. Together, these metrics characterize both translational and rotational accuracy, thereby enabling an objective comparison between the improved beaver behavior optimizer and the baseline beaver behavior optimizer for inverse kinematics solving on the rehabilitation mechanism.

**Figure 5:**
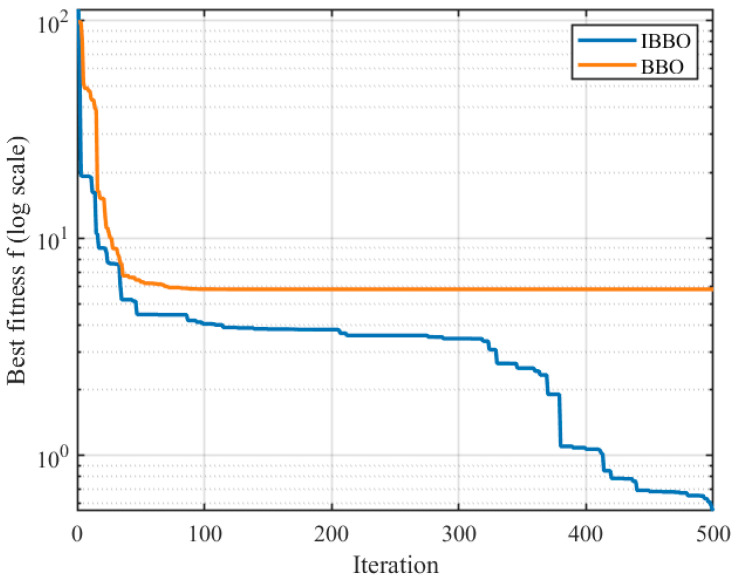
Comparison of convergence curves for inverse kinematics solving.

**Figure 6:**
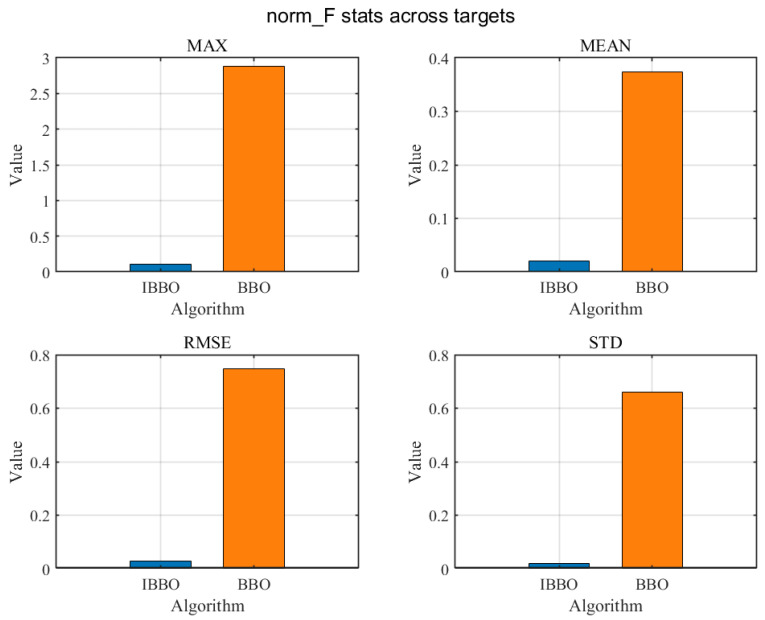
Comparison of Frobenius-norm errors.

**Figure 7:**
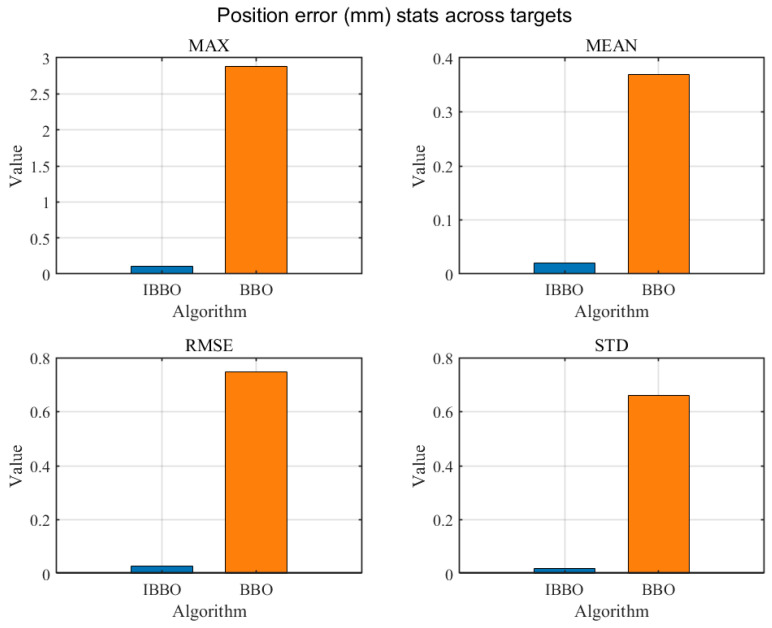
Comparison of position errors.

**Figure 8:**
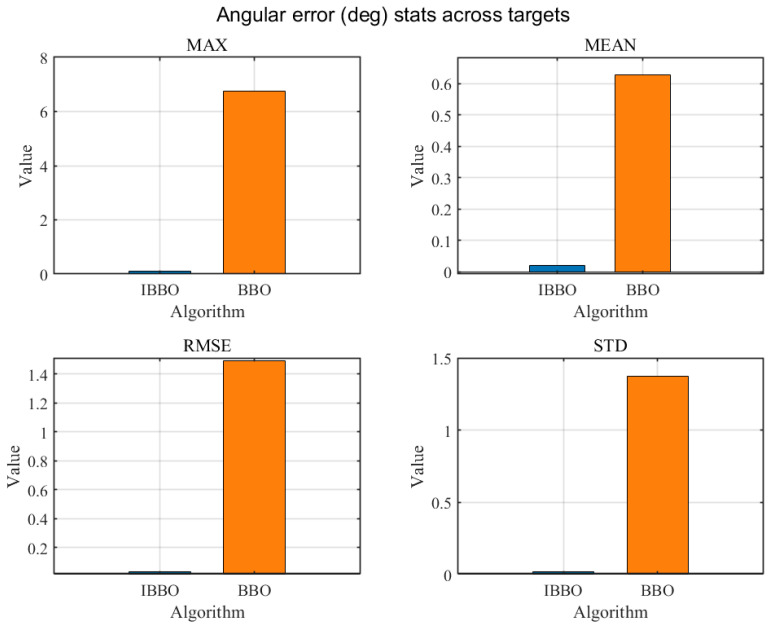
Comparison of orientation errors.

**Figure 9:**
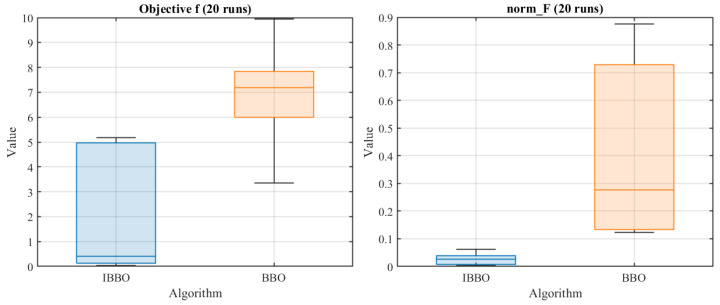

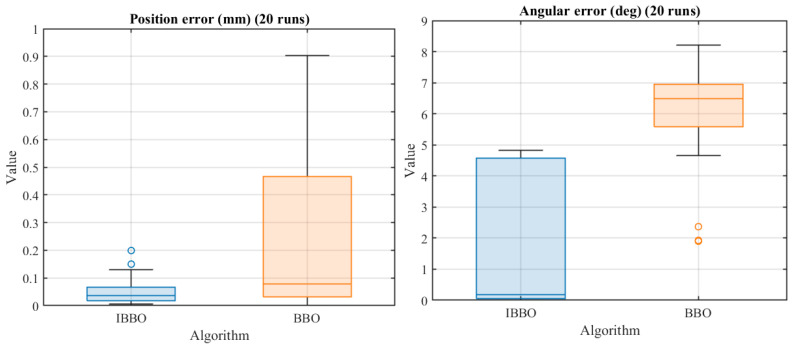
Boxplots of experimental results for the two algorithms.

**Figure 10:**
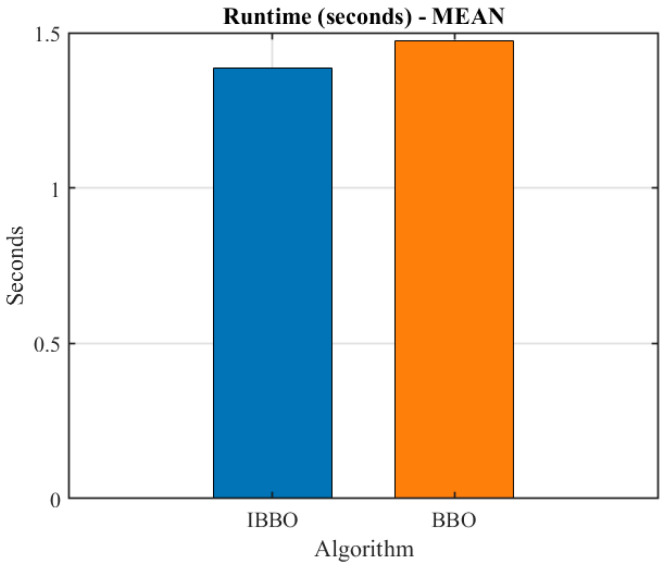
Runtime comparison between the two algorithms.

**Figure 11:**
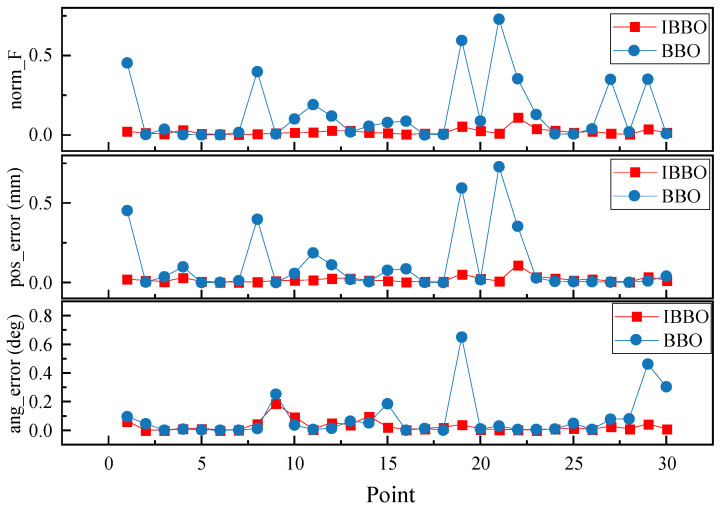
Target-point error comparison between the two algorithms.

Table 4 presents a pointwise comparison of the computed inverse-kinematics results obtained by the two algorithms for 10 arbitrary target end-effector pose points. Figure 5 then compares the convergence curves during inverse kinematics solving. Figure 6, Figure 7 and Figure 8 report the error comparisons from different perspectives: Figure 6 summarizes the Frobenius-norm errors of the pose transformation matrix, Figure 7 compares the position errors derived from the translational components, and Figure 8 compares the orientation (angular) errors derived from the rotational components. In addition to these pointwise and metric-wise evaluations, Figure 9 visualizes the distributions of the experimental outcomes using boxplots, covering the objective function value, Frobenius-norm error, position error, and angular error. Figure 10 compares the runtime of the two algorithms. Finally, Figure 11 provides a target-point-wise error comparison.

Table 4 compares the inverse-kinematics solutions obtained by IBBO and BBO for ten arbitrary target pose points using three complementary accuracy indicators: the Frobenius-norm error of the pose transformation, the position error, and the orientation error. The results show that the relative performance is target-dependent; however, IBBO exhibits clear advantages at several challenging points where BBO produces large deviations. For example, at Point 1 and Point 9, IBBO substantially reduces both norm_F and the position error, while also achieving lower overall pose discrepancies. IBBO also yields consistently competitive solutions on Points 3 and 10, with lower errors across all reported metrics. In contrast, BBO attains smaller errors on some targets (e.g., Points 5–8) in terms of both translational and rotational components, indicating that it can perform well on certain pose instances. Overall, Table 4 suggests that IBBO provides improved robustness against difficult target poses by mitigating large pose and position deviations, while maintaining comparable orientation accuracy on most points.

Figure 5 compares the convergence behaviors of IBBO and BBO for inverse kinematics solving by plotting the best-so-far objective value *f* versus iteration on a logarithmic scale. Both methods exhibit a rapid decrease in the early stage, indicating effective initial exploration. However, IBBO continues to achieve further improvements throughout the optimization process and maintains a consistently lower best fitness trajectory after the initial transient phase. In contrast, BBO quickly enters a long plateau at a higher fitness level, suggesting premature stagnation and limited refinement capability in later iterations. Notably, IBBO shows multiple stepwise reductions in the mid-to-late stage and eventually reaches a substantially lower terminal objective value, demonstrating stronger exploitation and better convergence quality. Overall, the convergence curves indicate that IBBO provides a more reliable search process for the inverse kinematics objective, achieving both faster progress after early iterations and a lower final solution cost than the baseline method.

Figure 6, Figure 7 and Figure 8 provide a statistical comparison of inverse-kinematics accuracy across the evaluated target poses by summarizing four descriptive indicators: Max, Mean, RMSE, and Std for the Frobenius-norm pose error, position error, and orientation error, respectively. As shown in Figure 6, the improved optimizer yields markedly smaller values for all four statistics of the Frobenius-norm error, indicating a substantially lower overall deviation of the predicted pose transformation matrix from the target and a reduced worst-case discrepancy. Consistently, Figure 7 demonstrates that the position errors produced by the improved method are significantly reduced in terms of Max, Mean, RMSE, and Std, implying both higher translational accuracy and improved robustness across different target points. A similar trend is observed for rotational performance in Figure 8, where the improved method achieves much smaller angular-error statistics, including a noticeably reduced maximum error and tighter dispersion, reflecting more reliable orientation matching. Collectively, these three figures confirm that the improved optimizer enhances inverse-kinematics solution quality in a comprehensive manner—simultaneously lowering typical errors (Mean/RMSE), suppressing outliers (Max), and improving consistency (Std) for both translation and rotation across the tested targets.

Figure 9 presents boxplot-based comparisons of the two algorithms over 20 independent runs in terms of the objective value *f*, the Frobenius-norm pose error (norm_F), the position error, and the angular error. Across all four metrics, the distributions associated with IBBO are concentrated at markedly lower levels than those of BBO, indicating consistently improved solution quality. IBBO exhibits a substantially lower median and a tighter interquartile range for both *f* and norm_F, suggesting not only better optimization outcomes but also stronger robustness with reduced run-to-run variability. Similar patterns are observed for the position and angular errors, where IBBO yields smaller central tendency and generally narrower spread, implying more reliable translational and rotational accuracy across repeated trials. By contrast, BBO shows higher medians and wider dispersion in all metrics, reflecting greater sensitivity to stochastic initialization and a higher likelihood of suboptimal convergence. Overall, the boxplots provide distributional evidence that IBBO improves both accuracy and stability for inverse kinematics solving on the rehabilitation mechanism.

Figure 10 compares the average runtime of the two algorithms for inverse kinematics solving. The results indicate that IBBO and BBO require comparable computational time, with both methods exhibiting mean runtimes on the order of approximately one second. This observation suggests that the accuracy and robustness improvements achieved by IBBO are not obtained at the expense of a substantial increase in computational cost. Therefore, IBBO provides a favorable performance–efficiency trade-off, making it suitable for practical inverse kinematics applications in rehabilitation robotics where both precision and runtime are important.

Figure 11 compares the target-wise error profiles of IBBO and BBO by plotting the Frobenius-norm pose error, position error, and angular error for each evaluated target point. Across the three subplots, IBBO maintains consistently low error levels with only minor fluctuations, indicating stable inverse-kinematics performance over different poses. In contrast, BBO exhibits pronounced error spikes at several points, leading to substantially larger norm_F and position errors and, in some cases, elevated angular errors. These peaks suggest that the baseline method is more sensitive to target-dependent difficulty and may occasionally converge to suboptimal solutions. Overall, the pointwise comparisons demonstrate that IBBO not only improves average accuracy but also effectively suppresses worst-case deviations, thereby providing more reliable pose tracking across a diverse set of target configurations.

Overall, the inverse-kinematics experiments consistently demonstrate the superiority of IBBO over the baseline BBO. IBBO converges to a lower objective value without premature stagnation, as reflected by the convergence curves. Across target poses, IBBO achieves markedly smaller Frobenius-norm, position, and orientation errors, with reduced Max, Mean, RMSE and Std statistics and tighter boxplot distributions, indicating improved accuracy and robustness. The target-wise comparisons further show that IBBO effectively suppresses error spikes that occur in BBO, yielding more stable performance across different poses. Importantly, these gains are obtained with a comparable mean runtime, confirming a favorable accuracy–efficiency trade-off for practical inverse-kinematics solving in rehabilitation robotics.

Although the current study evaluates solution quality mainly by the end-effector pose error, this criterion does not fully capture the practical requirements of rehabilitation robotics. In real rehabilitation scenarios, an optimal inverse kinematics solution should also consider patient comfort, motion safety, joint tolerance, and clinical appropriateness, many of which may rely on therapist experience or patient-specific feedback [22,23]. Therefore, the present work should be regarded as a numerical optimization framework for inverse kinematics, rather than a complete human-centered rehabilitation decision model. In future work, human feedback can be incorporated into the optimization process by introducing comfort-related penalty terms, safety-aware constraints, or clinician-evaluated preference scores, thereby extending the proposed IBBO framework toward human-in-the-loop rehabilitation optimization.

## 5. Conclusions

To address the persistent challenges of inverse kinematics in rehabilitation robotic arms, including strong nonlinearity, multiple feasible joint configurations, and strict joint-limit constraints, this study proposed an improved beaver behavior optimizer (IBBO) for optimization-based inverse kinematics solving. By incorporating strict elitist replacement with rollback, momentum-inspired information transfer, a lightweight memetic coordinate-wise local search, and an adaptive builder-disturbance schedule, the proposed method enhances the balance between exploration and exploitation while alleviating premature convergence. The effectiveness of IBBO was validated through both benchmark-function tests and inverse kinematics experiments on rehabilitation robotic arms. The main conclusions are summarized as follows.

(1) Competitive optimization capability and robustness. The benchmark results demonstrate that IBBO exhibits strong optimization performance, achieving competitive solution accuracy together with stable run-to-run behavior. These results indicate that the proposed method is a reliable metaheuristic solver for nonlinear and constrained optimization problems.
(2) Improved convergence performance in inverse kinematics optimization. In the inverse kinematics tasks, IBBO shows a more favorable convergence pattern than the baseline optimizer. It effectively avoids early stagnation and continues refining solutions toward lower objective values during the later search stages, indicating stronger exploitation capability and improved convergence quality.
(3) Higher pose accuracy with comparable computational efficiency. For rehabilitation-robot inverse kinematics, IBBO achieves lower pose errors, as reflected by reduced Frobenius-norm error, position error, and orientation error. In addition, it produces tighter error distributions and fewer target-dependent error spikes while maintaining a comparable mean runtime. This demonstrates that IBBO provides a better trade-off between solution accuracy and computational efficiency.

Overall, the results confirm that IBBO is an effective and robust optimization framework for solving inverse kinematics problems in rehabilitation robotic arms. Beyond its numerical advantages, the proposed method also shows the potential of translating beaver-inspired adaptive and cooperative behaviors into practical computational strategies for rehabilitation robotics.

Future work will focus on extending the proposed framework to more complex rehabilitation mechanisms and task scenarios, incorporating richer constraint models such as collision avoidance and dynamic feasibility, and exploring hybrid strategies with learning-based warm starts or adaptive parameter control to further improve generalization, stability, and real-time applicability.

## Figures and Tables

**Table 1:** Upper limb rehabilitation robot D-H parameters.

Joint *i*	Link Length *a_i_*/mm	Joint Offset *d_i_*/mm	Link Twist Angle *α_i_*/deg	Joint Angle *θ_i_*/deg
1	0	0	−90	*θ*_1_ ∈ [−90, 45]
2	0	0	−90	*θ*_2_ ∈ [−45, 90]
3	*a*_3_	0	0	*θ*_3_ ∈ [0, 140]
4	0	*d*_4_	−90	*θ*_4_ ∈ [−60, 30]
5	*a*_5_	0	−90	*θ*_5_ ∈ [−30, 30]

**Table 2:** Experimental results on the CEC 2022 (Dim = 20).

Function	Metric	PSO	PO	WOA	BBO	IBBO
F1	Ave	3.05304 × 10^3^	2.89714 × 10^4^	6.29064 × 10^4^	3.00324 × 10^2^	3.00143 × 10^2^
	Std	6.76512 × 10^3^	7.58679 × 10^3^	3.39375 × 10^4^	2.75830 × 10^−1^	6.53649 × 10^−2^
F2	Ave	1.54237 × 10^3^	9.88878 × 10^2^	4.69160 × 10^2^	4.58889 × 10^2^	4.55881 × 10^2^
	Std	4.82963 × 10^2^	1.85662 × 10^2^	2.81762 × 10^1^	1.21226 × 10^1^	1.18816 × 10^1^
F3	Ave	6.04006 × 10^2^	6.49557 × 10^2^	6.76798 × 10^2^	6.02051 × 10^2^	6.00405 × 10^2^
	Std	3.55187	8.55539	1.06468 × 10^1^	2.67648	1.15530
F4	Ave	8.40677 × 10^2^	9.46254 × 10^2^	9.66368 × 10^2^	8.39732 × 10^2^	8.21396 × 10^2^
	Std	1.18035 × 10^1^	1.58013 × 10^1^	2.33459 × 10^1^	1.27921 × 10^1^	6.15098
F5	Ave	9.96522 × 10^2^	3.03894 × 10^3^	3.48720 × 10^3^	1.02328 × 10^3^	9.00510 × 10^2^
	Std	1.68575 × 10^2^	6.95422 × 10^2^	7.98237 × 10^2^	1.30824 × 10^2^	6.33159 × 10^−1^
F6	Ave	4.47208 × 10^6^	3.20078 × 10^8^	6.48664 × 10^8^	4.51101e × 10^3^	3.66682e × 10^3^
	Std	1.31239 × 10^7^	1.22075 × 10^8^	8.15979 × 10^8^	3.10977 × 10^3^	1.39488 × 10^3^
F7	Ave	2.06033 × 10^3^	2.14170 × 10^3^	2.23363 × 10^3^	2.06585 × 10^3^3	2.05024 × 10^3^
	Std	3.38500 × 10^1^	2.86065 × 10^1^	6.47122 × 10^1^	3.63439 × 10^1^	1.25879 × 10^1^
F8	Ave	2.28720 × 10^3^	2.28581 × 10^3^	2.34168 × 10^3^	2.25941 × 10^3^	2.22541 × 10^3^
	Std	6.79818 × 10^1^	3.36396 × 10^1^	1.12329 × 10^2^	5.47716 × 10^1^	2.28352
F9	Ave	2.51849 × 10^3^	2.67595 × 10^3^	2.82493 × 10^3^	2.48085 × 10^3^	2.48083 × 10^3^
	Std	4.78258 × 10^1^	4.88987 × 10^1^	1.07099 × 10^2^	6.26016 × 10^−2^	3.57038 × 10^−2^
F10	Ave	3.14269 × 10^3^	3.49186 × 10^3^	6.19334 × 10^3^	3.01343 × 10^3^	2.55368 × 10^3^
	Std	5.04244 × 10^2^	1.65755 × 10^3^	1.17447 × 10^3^	4.36370 × 10^2^	1.91182 × 10^2^
F11	Ave	2.90000 × 10^3^	3.05014 × 10^4^	8.90454 × 10^3^	2.90417 × 10^3^	2.90245 × 10^3^
	Std	1.35814 × 10^−11^	7.48845 × 10^3^	1.26489 × 10^3^	2.43573	6.34413
F12	Ave	3.01848 × 10^3^	3.12555 × 10^3^	3.29696e × 10^3^	2.96383 × 10^3^	2.94768 × 10^3^
	Std	5.38569 × 10^1^	3.66788 × 10^1^	1.84033 × 10^2^	2.25913 × 10^1^	6.87448

**Table 3:** Summary of statistical significance tests and ranking results on the CEC 2022 (Dim = 20).

Function	Metric	PSO	PO	WOA	BBO	IBBO
F1	F-ranking	3	4	5	2	1
	Wilcoxon	(+)	(+)	(+)	(+)	—
	*p*-value	*p* < 0.05	*p* < 0.05	*p* < 0.05	*p* < 0.05	—
F2	F-ranking	3	4	5	2	1
	Wilcoxon	(+)	(+)	(+)	(+)	—
	*p*-value	*p* < 0.05	*p* < 0.05	*p* < 0.05	*p* < 0.05	—
F3	F-ranking	3	4	5	2	1
	Wilcoxon	(+)	(+)	(+)	(+)	—
	*p*-value	*p* < 0.05	*p* < 0.05	*p* < 0.05	*p* < 0.05	—
F4	F-ranking	3	4	5	2	1
	Wilcoxon	(+)	(+)	(+)	(+)	—
	*p*-value	*p* < 0.05	*p* < 0.05	*p* < 0.05	*p* < 0.05	—
F5	F-ranking	2	4	5	3	1
	Wilcoxon	(+)	(+)	(+)	(+)	—
	*p*-value	*p* < 0.05	*p* < 0.05	*p* < 0.05	*p* < 0.05	—
F6	F-ranking	3	4	5	2	1
	Wilcoxon	(+)	(+)	(+)	(+)	—
	*p*-value	*p* < 0.05	*p* < 0.05	*p* < 0.05	*p* < 0.05	—
F7	F-ranking	2	4	5	3	1
	Wilcoxon	(+)	(+)	(+)	(+)	—
	*p*-value	*p* < 0.05	*p* < 0.05	*p* < 0.05	*p* < 0.05	—
F8	F-ranking	2	4	5	3	1
	Wilcoxon	(+)	(+)	(+)	(+)	—
	*p*-value	*p* < 0.05	*p* < 0.05	*p* < 0.05	*p* < 0.05	—
F9	F-ranking	3	4	5	2	1
	Wilcoxon	(+)	(+)	(+)	(+)	—
	*p*-value	*p* < 0.05	*p* < 0.05	*p* < 0.05	*p* < 0.05	—
F10	F-ranking	3	4	5	2	1
	Wilcoxon	(+)	(+)	(+)	(+)	—
	*p*-value	*p* < 0.05	*p* < 0.05	*p* < 0.05	*p* < 0.05	—
F11	F-ranking	1	5	4	3	2
	Wilcoxon	(-)	(+)	(+)	(+)	—
	*p*-value	*p* < 0.05	*p* < 0.05	*p* < 0.05	*p* < 0.05	—
F12	F-ranking	3	4	5	2	1
	Wilcoxon	(+)	(+)	(+)	(+)	—
	*p*-value	*p* < 0.05	*p* < 0.05	*p* < 0.05	*p* < 0.05	—

**Table 4:** Comparison of the results of the two algorithms for 10 arbitrary target pose points.

Point	IBBO			BBO		
	norm_F	pos_error (mm)	ang_error (deg)	norm_F	pos_error (mm)	ang_error (deg)
1	0.0225	0.0225	0.0631	0.4532	0.4532	0.0351
2	0.0128	0.0128	0.0010	0.0035	0.0033	0.0461
3	0.0068	0.0068	0.0004	0.0369	0.0369	0.0008
4	0.0314	0.0314	0.0128	0.0259	0.0199	0.0151
5	0.0079	0.0079	0.0134	0.0011	0.0052	0.0004
6	0.0030	0.0030	0.0013	0.0018	0.0018	0.0007
7	0.0370	0.03746	0.0044	0.0156	0.0011	0.0041
8	0.0263	0.0263	0.0074	0.0223	0.0223	0.0019
9	0.1103	0.1103	0.0047	0.3538	0.3538	0.0064
10	0.0154	0.0154	0.0157	0.0262	0.0261	0.0488

## Data Availability

All data generated and analyzed during this study are included in this published article. The MATLAB implementation of the Improved Beaver Behavior Optimizer (IBBO) is publicly available at: https://github.com/DYHuestc/IBBO (accessed on 18 March 2026).
